# High-mobility group box-1 induces vascular remodelling processes *via* c-Jun activation

**DOI:** 10.1111/jcmm.12519

**Published:** 2015-02-28

**Authors:** Diana Zabini, Slaven Crnkovic, Hui Xu, Maria Tscherner, Bahil Ghanim, Walter Klepetko, Andrea Olschewski, Grazyna Kwapiszewska, Leigh M Marsh

**Affiliations:** a Ludwig Boltzmann Institute for Lung Vascular ResearchGraz, Austria; b Experimental Anaesthesiology, Department of Anaesthesia and Intensive Care Medicine, Medical University of GrazGraz, Austria; c Division of Pulmonology, Department of Internal Medicine, Medical University of GrazGraz, Austria; d Division of Thoracic Surgery, Department of Surgery, Medical University of ViennaVienna, Austria

**Keywords:** alarmins, HMGB1, inflammation, proliferation, pulmonary hypertension

## Abstract

Extracellular high-mobility group box-1 (HMGB1) acts as a signalling molecule during inflammation, cell differentiation and angiogenesis. Increased abundance of HMGB1 is associated with several pathological disorders such as cancer, asthma and chronic obstructive pulmonary disease (COPD). In this study, we investigated the relevance of HMGB1 in the pathological remodelling present in patients with idiopathic pulmonary arterial hypertension (IPAH) and pulmonary hypertension (PH) associated with COPD. Remodelled vessels present in COPD with PH and IPAH lung samples were often surrounded by HMGB1-positive cells. Increased HMGB1 serum levels were detected in both patient populations compared to control samples. The effects of physiological HMGB1 concentrations were then examined on cellular responses *in vitro*. HMGB1 enhanced proliferation of pulmonary arterial smooth muscle cells (PASMC) and primary human arterial endothelial cells (PAEC). HMGB1 stimulated p38, extracellular signal-regulated kinase (ERK) and c-Jun N-terminal kinase (JNK) phosphorylation. Furthermore, activation of the downstream AP-1 complex proteins c-Fos and c-Jun was observed. Silencing of c-Jun ablated the HMGB1-induced proliferation in PASMC. Thus, an inflammatory component such as HMGB1 can contribute to PASMC and PAEC proliferation and therefore potentially to vascular remodelling and PH pathogenesis.

## Introduction

Alarmins are a diverse family of unrelated molecules that possess important intracellular roles, however, they can also be released from cells during necrosis or in response to infection 1. Known alarmins include the S100 family, high-mobility group box-1 (HMGB1) and heat shock proteins. HMGB1 is a non-histone chromosomal protein that acts to stabilize nucleosome formation and regulate transcription. HMGB1 has an important role extracellularly in mediating the inflammatory response, acting not only as an endogenous danger signal but also as a chemoattractant 2. *In vivo*, HMGB1 has been shown to be released by damaged or necrotic cells, including endothelial cells and activated macrophages, as a cytokine mediator of inflammation 3. When released extracellular HMGB1 binds to its receptors, advanced glycation end products (RAGE) or toll like receptors (TLR). HMGB1 has been implicated in several pathological conditions including rheumatic disease, sepsis and myocardial infarction. Increased levels of HMGB1 in the sputum or bronchoalveolar lavage fluid of asthma, chronic obstructive pulmonary disease (COPD), systemic sclerosis and idiopathic pulmonary fibrosis patients are associated with disease severity 4–8.

Pulmonary hypertension (PH) is a life-threatening disease characterized by progressive remodelling of the pulmonary arteries and increased pulmonary arterial pressure. Hallmarks of vascular remodelling include thickening of the intima, media and adventitia 9. PH can be associated with autoimmune and infectious diseases, *e.g*. connective tissue diseases 10,11, HIV 12 and schistosomiasis 13. The presence of PH in patients with connective tissue diseases correlates with poorer prognoses and worse outcome 10,14,15. An inflammatory component is present in interstitial lung diseases, COPD as well as in idiopathic pulmonary arterial hypertension (IPAH) 16,17. In diseases associated with inflammatory conditions an increased incidence of PAH has been observed (reviewed in Ref. 18). In PAH-affected human lungs, an accumulation of inflammatory cells including antigen-presenting cells (macrophages, dendritic cells) and effector cells (T cells) has been described 19,20. Recently, Stacher *et al*. demonstrated that the increased perivascular inflammation observed in PAH lungs correlated with intima and media remodelling 17. However, how these cells contribute to disease pathogenesis is still poorly understood. We aim to investigate the role of HMGB1 in human vascular disease and its effects on vascular remodelling processes.

## Materials and methods

### Patient samples

Lung tissues were obtained from three IPAH patients and three patients with PH associated with COPD (COPD+PH), who had undergone lung transplantation at the Division of Thoracic Surgery, Medical University of Vienna, Austria. Non-transplanted donor lungs (*n* = 3) that had been harvested for transplantation, but not implanted because of size-reduced lung transplantation, served as controls. Samples were fixed in 4% (m/v) formaldehyde. Human serum from IPAH and COPD+PH patients (*n* = 14 per group) was collected during right heart catheterization or at the time of lung transplantation. In addition, serum was taken from 14 healthy volunteers and stored at −80°C until use. All studies were approved by the ethical committees for the Medical Universities of Graz and Vienna, and written informed consent obtained from all study participants. Patient data are listed in Table1 (mean ± SD).

**Table 1:** Patient parameters for serum samples (mean ± SD)

	Controls	IPAH	COPD+PH
Sex (female:male)	12:2	12:2	7:7
Age (years)	64.5 ± 11.2	64.2 ± 10.0	61.4 ± 8.8
mPAP (mmHg)		42.4 ± 11.8	35.7 ± 11.0

IPAH, idiopathic pulmonary arterial hypertension; COPD+PH, pulmonary hypertension associated with chronic obstructive pulmonary disease; mPAP, mean pulmonary arterial pressure.

### Immunostaining

Immunohistochemical (IHC) staining was performed on 3-μm thick paraffin-embedded lung sections. Sections were deparaffinized in xylene followed by decreasing concentrations of ethanol before rehydration in PBS. Antigen retrieval was performed with 0.05% trypsin (Zymed Laboratories, Thermo Fisher Scientific, Waltham, MA, USA) for 10 min. at 37°C. Tissue was blocked for 1 hr with 10% bovine serum albumin (BSA) followed by double IHC staining against alpha smooth muscle actin (alpha-SMA; 1:200; Everest Biotech Ltd, Oxford, UK) and von Willebrand factor (vWF, 1:900; Dako, Glostrup, Denmark) to detect smooth muscle and endothelial cells respectively. ImmPRESS α-Goat Ig (peroxidase) Polymer detection kit together with the VECTOR VIP substrate kit and the ImmPRESS α-Rabbit Ig (peroxidase) with DAB (all from Vector Laboratories, Burlingame, CA, USA); counterstaining was performed with methyl green. For CD45 (1:200), HMGB1 (1:1000), TLR4 (1:50) and RAGE (1:400) staining (all antibodies from Abcam, Cambridge, UK), antigen retrieval sodium citrate buffer (Dako) was used for 20 min. at 95°C. Serum block (Vector Laboratories) was used for 20 min. Primary antibodies were detected with ImmPRESS α-Rabbit Ig (peroxidase) together with Nova Red or DAB substrate kit (Vector Laboratories). Counterstaining was performed with haematoxylin. Control stainings were performed with the omission of the primary antibody. Whole slide images were made using an Aperio Scanscope slide scanner (Aperio, Oxford, UK).

### ELISA

The concentration of HMGB1 was determined in human serum samples according to the manufacturer's instructions (IBL International, Hamburg, Germany).

### Isolation, culture of human pulmonary artery smooth muscle cells and pulmonary arterial endothelial cells

Human primary pulmonary arterial smooth muscle cells (PASMC) were isolated from resistance arteries less than 1 mm in diameter obtained from non-utilized human donor lungs (*n* = 10) and cultured in VascuLife® SMC Medium (LifeLine Technology, Walkersville, MD, USA). The adventitia and endothelial layers were removed by mechanical separation. The remaining tissue was cut into small pieces and cultured to allow the outgrowth of PASMC. The purity of PASMC preparations was confirmed using immunofluorescence staining against smooth muscle myosine heavy chain (1:50; Abcam) and alpha-SMA (1:300; Everest Biotech Ltd); minimum 95% of cells stained positive; Figure S1. The immunofluorescence staining was performed on 4% PFA-fixed cells and Alexa 555-labelled secondary antibodies were applied (Life Technologies, Thermo Fisher Scientific, Waltham, MA, USA). Human PASMC between passages 3 and 6 were used for the experiments. Primary human arterial endothelial cells (PAEC) were purchased from Lonza (Basel, Switzerland) (*n* = 6), cultured in VascuLife® VEGF Medium (LifeLine Technology, Frederick, MD, USA) and used for experiments until passage 8. Cells were incubated at 37°C in a humidified atmosphere of 5% CO_2_.

### Proliferation assay

To investigate the effect of HMGB1 on PASMC and PAEC proliferation, the following protocol was applied: 10,000 PAEC or 5000 PASMC were seeded in 96-well plates. Prior to stimulation, PASMC were kept in quiescent medium [VascuLife® basal medium with 1% penicillin/streptomycin (P/S) and 0.1% foetal calf serum (FCS)] for 24 hrs. Cells were then treated for 24 hrs with 1 or 100 ng/ml HMGB1 (fully reduced; LPS-free HMGB1 HMGbiotech, IBL International) or were kept under control conditions (vehicle/PBS). To investigate the effect of specific MAP kinases on HMGB1-induced PASMC and PAEC proliferation, cells were pre-incubated after the 24 hrs starvation step either with ERK (50 μM U0126), JNK (5 μM SP600125), p38 (8 μM SB203580) inhibitors (all from Sigma-Aldrich, Taufkirchen, Germany) or vehicle control Dimethyl sulfoxide (DMSO). After 1 hr pre-incubation with inhibitors, media was removed and replaced with 1 or 100 ng/ml HMGB1 for 24 hrs, or kept under control conditions (vehicle/PBS). To assess the effect of c-Jun silencing on HMGB1-induced PASMC proliferation, 150,000 cells were seeded in six-well plates. The following day, the media was replaced with basal media 0% FCS for 2 hrs and cells transfected with 100 nM siRNA against c-Jun (On Target plus Smart pool, Thermo Scientific, Wilmington, DE, USA) or non-silencing control siRNA (Thermo Scientific). After 6 hrs, medium was changed to full medium. The next day, 5000 cells/well were seeded in 96-well plates and starved overnight in quiescent media before treatment with 1 or 100 ng/ml HMGB1 for 24 hrs. The proliferation of PASMC and PAEC was determined by [3H]-thymidine (Biotrend Chemikalien GmbH, Cologne, Germany) incorporation as an index of DNA synthesis and radioactivity was measured by a scintillation counter (Wallac 1450 MicroBeta TriLux Liquid Scintillation Counter & Luminometer, Perkin Elmer, Monza, Italy). Experiments were performed in quintuplicate and repeated 5–10 times.

### Apoptosis

PAEC and PASMC were grown until confluent in six-well dishes and treated for 24 hrs either with 0, 1 or 100 ng/ml HMGB1. Prior to stimulation, PASMC were kept in quiescent medium for 24 hrs, whereas PAEC were grown in full medium (VascuLife® VEGF Medium). Apoptotic cell death was determined by Annexin V–FITC/propidium iodide (PI) staining (FITC Annexin V Apoptosis Detection Kit I, BD Biosciences, San Diego, CA, USA) according to the manufacturer's instructions. Experiments were performed five times with PAEC and PASMC. Staurosporin 0.05 μM (Cayman Europe, Tallinn, Estonia) was used as a positive control.

### Cell adhesion

Cell culture plates (96-well) were coated overnight with 5 μg/ml fibronectin at 4°C and then blocked with 3% BSA in PBS for 1 hr at room temperature (RT). Freshly trypsinized PAEC and PASMC were incubated with 0, 1 or 100 ng/ml HMGB1 for 20 min. at RT in basal medium (0% FCS). Cells were then seeded at a density of 20,000 cells per well in basal medium and left to adhere for 45 min. at 37°C. Wells were washed with PBS and cells fixed with ice-cold methanol–acetone (1:2) for 20 min. at 4°C, before staining with crystal violet solution (0.5% crystal violet (w/v), 20% methanol, 80% ddH_2_O). Cells were washed with H_2_O, then dried before destaining with 30% methanol, 10% acetic acid and 60% ddH_2_O. Absorbance was measured at 590 nm.

### Migration assay

Cell migration assays were performed with transwell chambers (24-well, 8-μm pore size, Falcon, Corning Life Sciences, Amsterdam, The Netherlands) in duplicate. Growth medium containing 0.1% FCS (PASMC) or 1% FCS (PAEC) supplemented with 0, 1 or 100 ng/ml HMGB1 was added to the lower chamber. PAEC and PASMC were seeded in same growth medium as above without HMGB1 at a density of 20,000 in the upper chamber and allowed to migrate for 16 hrs. Transwells were then washed with PBS, fixed and stained with crystal violet solution for 20 min., before washing with H_2_O. Cells on the lower side of the filter were counted under a microscope.

### RNA isolation and real-time PCR

Total cellular RNA from PAEC and PASMC was isolated using the RNeasy Mini Kit (Qiagen, Hilden, Germany). A Nanodrop 2000c spectrophotometer (Thermo Scientific) was used to quantify the concentration and the purity of the isolated total RNA. Total RNA was reverse transcribed to cDNA using an iScript™ cDNA Synthesis Kit (Bio-Rad, Hercules, CA, USA) and amplified using a Lightcycler 480 (F. Hoffman-La Roche, Basel, Switzerland). The PCR reactions were set up using QuantiFast SYBR PCR kit (Qiagen). Cycling conditions were as follow: 5 min. at 95°C (5 sec. at 95°C, 5 sec. at 60°C, and 10 sec. at 72°C) ×45. Melting curve analysis and gel electrophoresis were performed to confirm the specific amplification of the expected PCR product. Gene expression was calculated using the ΔCt method using porphobilinogen deaminase (PBGD) and beta-2-microglobulin (B2M) as reference genes. Primer sequences are listed in Table2.

**Table 2:** Primer sequences used in the study

Gene	Accession number	Forward primer (5′–3′)	Reverse primer (5′–3′)
B2M	NM_004048.2	CCTGGAGGCTATCCAGCGTACTCC	TGTCGGATGGATGAAACCCAGACA
Col1a1	NM_000088.3	ACATGTTCAGCTTTGTGGACC	TGTACGCAGGTGATTGGTGG
Col3a1	NM_000090	GGTGTCCCAGGGAAAGATGG	CTCTCTCACCAGGGCTACCA
MMP1	NM_002421.3	AGTCCAGAAATACCTGGAAAAATAC	TTTTTCAACCACTGGGCCAC
MMP10	NM_002425.2	TCCAGGAGTTGAGCCTAAGGT	CGCCTAGCAATGTAACCAGC
MMP19	NM_002429.5	GCCCGTGGACTACCTGTCAC	TGTGGCATCATCCAGCTGAC
PBGD	NM_000190.3	CCCACGCGAATCACTCTCAT	TGTCTGGTAACGGCAATGCG
RAGE	NM_001136.4	GCCACTGGTGCTGAAGTGTA	TCCGGCCTGTGTTCAGTTTC
TIMP1	NM_003254.2	GCCTTCTGCAATTCCGACCT	TTGGAACCCTTTATACATCTTGGT
TIMP2	NM_003255.4	ATGCAGATGTAGTGATCAGGGC	TCTATATCCTTCTCAGGCCCTTTG
TIMP3	NM_000362.4	CTCCGACATCGTGATCCGGG	TGGATGTACTGCACATGGGG
TLR4	NM_003266.3	AAGAGCTGGCATGAAACCCA	ATTAGGAACCACCTCCGTGA
TnC	NM_002160	GTCAAGCAACCCAGCCAAAG	TCTGTCTGGGAAACACGTCG

### Protein isolation and Western blotting

Primary human arterial endothelial cells and PASMC were lysed in RIPA buffer (Sigma-Aldrich) and 10 μg of protein were run on a 10% SDS polyacrylamide gel, followed by electrotransfer to an Amersham Hybond P 0.45 PVDF Blotting Membrane (GE Healthcare, Wien, Austria). After blocking with 5% non-fat dry milk in TBS-T buffer (Tris-buffered saline with 0.1% Tween-20), the membrane was incubated overnight at 4°C with one of the following antibodies: anti-p38, anti–phospho-p38 (T180/Y182), anti-c-Jun; anti-phospho c-Jun (S73), anti-phospho Erk1/2 (T202/Y204), anti-Erk1/2, anti-phospho c-Fos (S32) all from Cell Signaling and anti-c-Fos (Novus Biologicals, Cambridge, UK) were used at dilution of 1:1000; and anti-TLR4 (Abcam), anti-RAGE (1:500; Abcam) and rabbit anti–alpha-tubulin (1:5000; Cell Signaling, Boston, MA, USA). After washing, the membranes were incubated with horseradish-peroxidase–labelled secondary antibodies (1:5000, anti-rabbit-HRP, Pierce, Thermo Fisher Scientific, Waltham, MA, USA) and proteins detected with Amersham ECL Prime Western Blotting Detection System (GE Healthcare). Antibodies were removed by incubating the membrane for 15 min. with stripping buffer (RestoreTM PLUS Western Blot Stripping Buffer; Thermo Scientific). Densitometric analysis was performed with ImageJ (National Institutes of Health, USA).

### Statistical analysis

Graphing and statistical analysis was performed with Graph Pad Prism 5 (La Jolla, CA, USA). Data are expressed as mean ± SEM or as mean and percentiles. Data from multiple groups were compared for statistical significance using the Kruskal–Wallis test or repeated measures anova with Dunnett's multiple-to-one comparison test. Paired *t*-test was used to compare two groups. Statistical significance was referred to as follows: *P ≤ 0.05, **P ≤ 0.01, ***P ≤ 0.001.

## Results

### HMGB1 in human pulmonary disease

**Figure 1:**
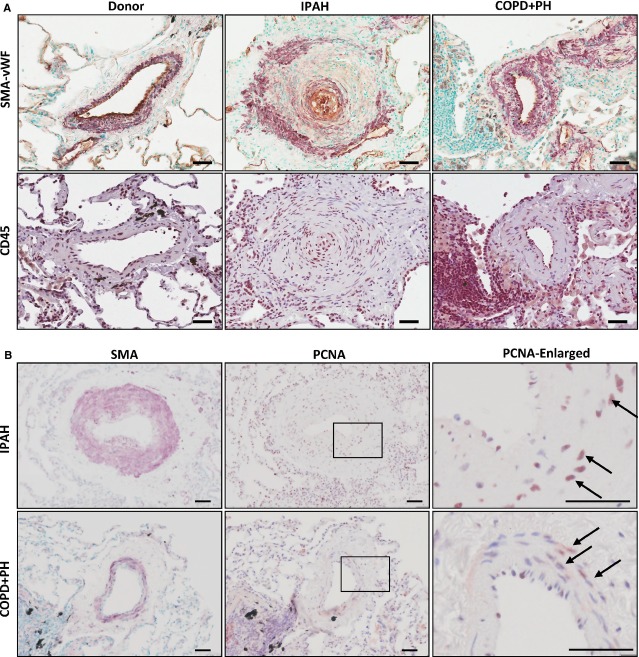
Vascular remodelling and inflammatory cell localization in IPAH- and COPD-associated PH lung tissue. (A) Representative images of lung sections from donor, idiopathic pulmonary arterial hypertension (IPAH, *n* = 3) or PH associated with chronic obstructive pulmonary disease (COPD+PH, *n* = 3) patients stained against: (upper panels) smooth muscle actin (SMA, purple) and von Willebrand (vWF, brown) and CD45 (lower panels); (B) Representative images of proliferating cell nuclear antigen (PCNA) staining in IPAH and COPD+PH patients. Higher scale images (right panels) show boxed area in centre panels. Arrows indicate positive nuclei. SMA staining is shown to visualize the vessel wall, Scale bars represent 50 μm.

As a result of the proposed inflammatory component in human PH, we first examined remodelling and the presence of inflammatory cells in IPAH patients and also in patients with PH associated with COPD (COPD+PH). The remodelled vessels of IPAH and COPD+PH patients were associated with inflammatory cells as shown by CD45 positive staining (Fig.1A). Concurrent with the vascular remodelling observed in IPAH and COPD+PH samples, a number of proliferating cells were detected in the vessel medial layer (Fig.1B).

**Figure 2:**
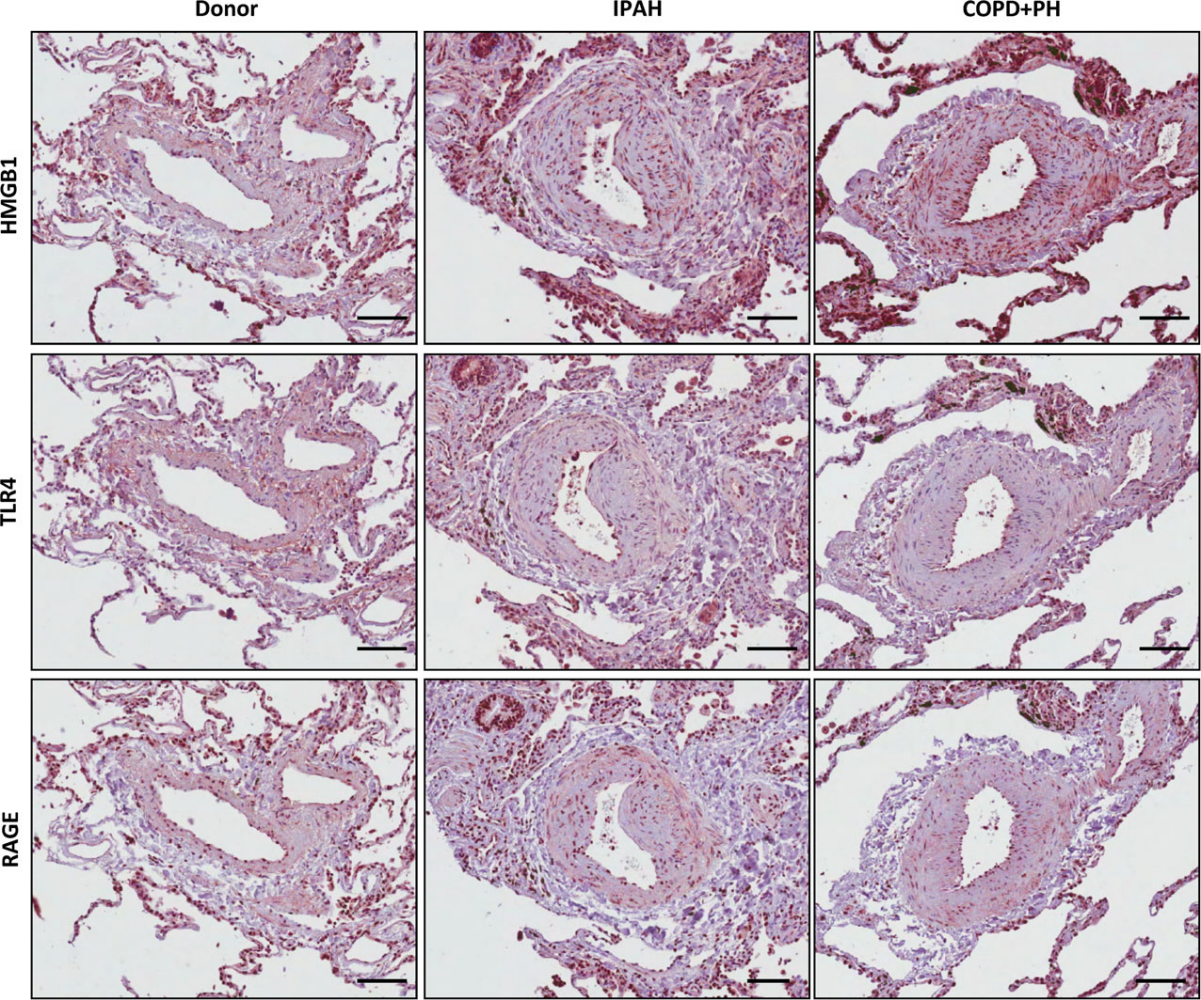
Localization of HMGB1 and receptors in IPAH- and COPD-associated PH lung tissue. Immunohistochemical staining for HMGB1 (upper panels), TLR4 (middle panels) and RAGE (lower panels) in lung sections from donor, idiopathic pulmonary arterial hypertension (IPAH) or PH associated with chronic obstructive pulmonary disease (COPD+PH). Scale bars represent 50 μm.

**Figure 3:**
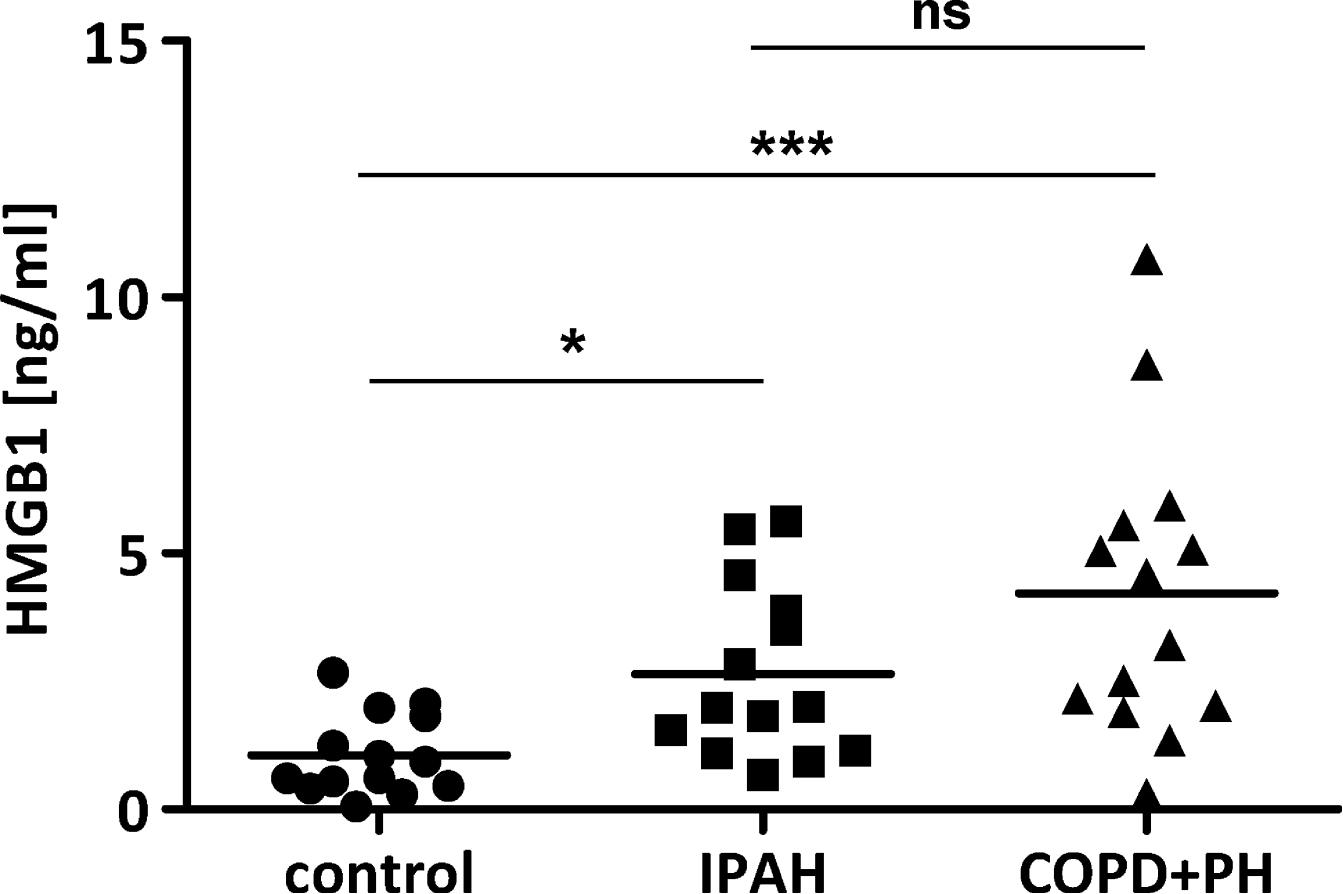
Increased serum levels of HMGB1 in IPAH- and COPD-associated PH patients. Serum levels of HMGB1 in donors, idiopathic pulmonary arterial hypertension (IPAH) or PH associated with chronic obstructive pulmonary disease (COPD+PH) patients; *P < 0.05, ***P < 0.001, ns not significant.

Staining against HMGB1 in healthy donor lung sections demonstrated moderate immunoreactivity in the bronchial epithelium and alveolar macrophages. In sections from COPD+PH and IPAH explant lungs, strong immunoreactivity was visible in inflammatory cells. Weaker immunoreactivity was observed in the endothelial and smooth muscle layers. Donor samples exhibited weaker HMGB1 staining compared to diseased tissue (Fig.2). In contrast, receptors for HMGB1 (TLR4 and RAGE) were widely expressed throughout the entire lung tissue in both disease and control lungs (Fig.2). As the high nuclear expression of HMGB1 prevents investigating expression changes by real-time PCR or Western blotting, we analysed extracellular HMGB1 levels in the serum of patient populations. Serum levels of HMGB1 were significantly elevated in both IPAH and COPD+PH samples as compared to controls (2.6 ± 0.45 ng/ml, *n* = 14; 4.2 ± 0.78 ng/ml, *n* = 14; *versus* 1.05 ± 0.2 ng/ml, *n* = 14, respectively; Fig.3).

### HMGB1 induces proliferation in PASMC and PAEC

**Figure 4:**
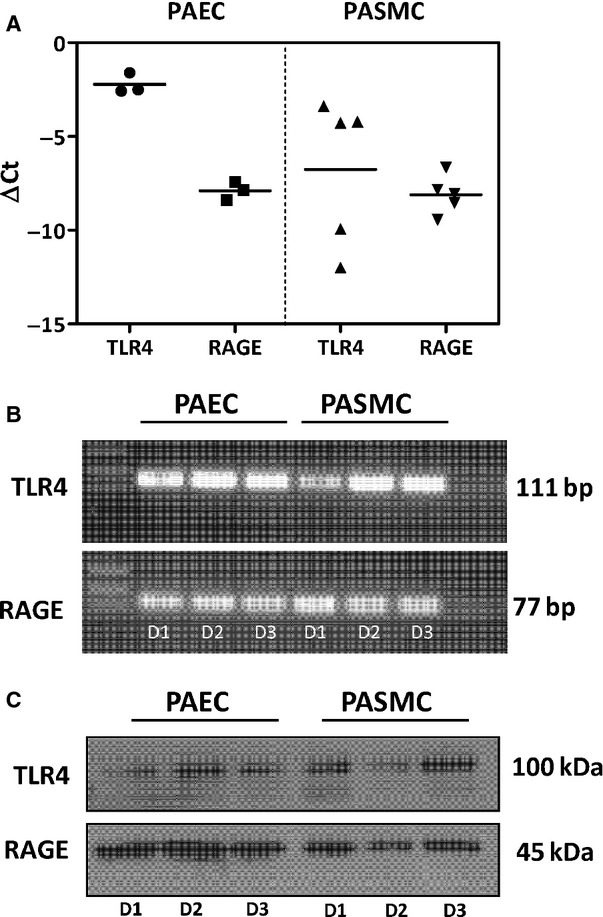
Human pulmonary arterial endothelial cells and smooth muscle cells express the HMGB1 receptors TLR4 and RAGE. Relative TLR4 and RAGE mRNA expression (A and B) and protein (C) expression in human pulmonary arterial endothelial cells (PAEC) and human pulmonary arterial smooth muscle cells (PASMC); D1–D3 represent samples from three different donors for PASMC or lots for PAEC.

**Figure 5:**
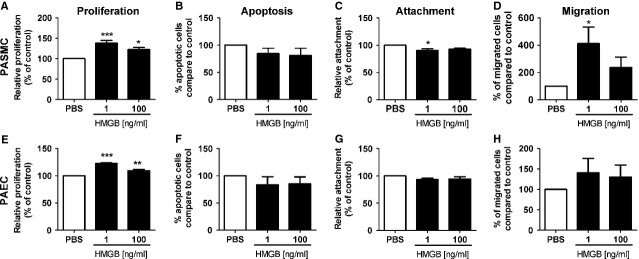
HMGB1-induced proliferation of pulmonary arterial endothelial cells and smooth muscle cells. Effect of HMGB1 treatment on human pulmonary arterial smooth muscle cells (PASMC, A–D) and human pulmonary arterial endothelial cells (PAEC, E–H) on (A and E) proliferation *n* = 5–6, (B and F) apoptosis *n* = 5, (C and G) attachment *n* = 5 and (D and H) migration *n* = 4–6. *P < 0.05, **P < 0.01, ***P < 0.001.

As a result of the strong IHC staining and raised circulatory levels of HMGB1 in the patient samples (Figs1 and 3), we sought to investigate the effects of HMGB1 stimulation on vessel cell homoeostasis. We first confirmed that the HMGB1 receptors, TLR4 and RAGE, were expressed by both PASMC and PAEC. Real-time PCR and Western blotting revealed that both TLR4 and RAGE were present in PASMC and PAEC (Fig.4). Treatment of PASMC and PAEC with either 1 or 100 ng/ml HMGB1 resulted in enhanced proliferation, as demonstrated by increased thymidine incorporation (Fig.5A and E). Analysis of apoptosis revealed no changes between HMGB1- or control-treated cells (Fig.5B and F). HMGB1 at 1 ng/ml caused a slight reduction in PASMC attachment on fibronectin-coated plates and increased PASMC cell migration (Fig.5C–H) without an associated effect on gene expression of extracellular matrix components (Fig. S2).

### HMGB1 induces c-Fos/c-Jun activation in PASMC

**Figure 6:**
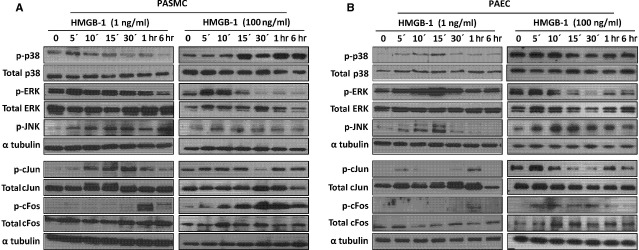
HMGB1 activates MAPK intracellular signalling pathways. Western blot analysis for MAPK and downstream factor activation in (A) human pulmonary arterial smooth muscle cells (PASMC) and (B) human pulmonary arterial endothelial cells (PAEC) following stimulation with 1 or 100 ng/ml HMGB1 for the indicated time-points. Blots are representative of a minimum three independent experiments.

We next focused on which intracellular signalling pathways are activated in response to HMGB1. In PASMC, low concentrations (1 ng/ml) of HMGB1 caused a time-dependent phosphorylation of the p38 and JNK pathways. At higher concentrations (100 ng/ml), additional activation of ERK was observed (Fig.6A and Fig. S3). At both high and low concentrations, HMGB1 activated the ERK pathway in PAEC, however, a robust activation of JNK was only observed at 100 ng/ml and p38 activation at 1 ng/ml (Fig.6B and Fig. S3). Downstream from MAPK, both HMGB1 concentrations increased c-Jun and c-Fos protein phosphorylation at later time-points in PASMC. However, no significant activation of c-Jun was observed in PAEC (Fig.6 and Fig. S3).

### HMGB1 induced proliferation is dependent on JNK and c-Jun

**Figure 7:**
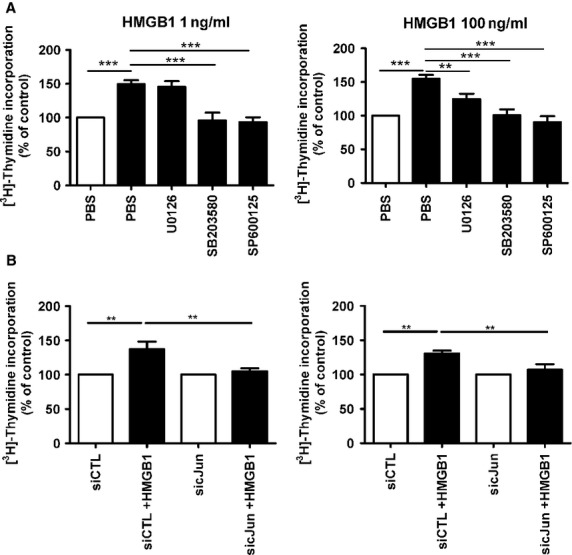
Silencing of c-Jun attenuates HMGB1-induced proliferation. (A) Effects of MAPK inhibition of HMGB1-induced PASMC proliferation as determined by relative thymidine incorporation with prior treatment with ERK1/2 (U0126), p38 (SB203580), JNK (SP600125) inhibitors, *n* = 6. (B) Effects of siRNA-mediated c-Jun silencing (sicJun) on HMGB1-induced PASMC proliferation, siCTL (control siRNA), *n* = 6. **P < 0.01, ***P < 0.001.

As strong MAPK activation was observed in response to HMGB1 stimulation, we examined whether the use of specific MAPK inhibitors could prevent the pro-proliferative effects of HMGB1 in PASMC. Inhibition of the JNK (SP600125) and p38 (SB203580) pathways fully attenuated the proliferation of PASMC induced by both low and high HMGB1 concentrations. In contrast, inhibition of ERK1/2 (U0126) only reduced PASMC proliferation at 100 ng/ml HMGB1 (Fig.7A). As both JNK and its downstream transcription factor c-Jun were activated in PASMC, we investigated whether knockdown of c-Jun could also reduce HMGB1-driven proliferation. This revealed that silencing of c-Jun significantly attenuated the HMGB1-induced PASMC proliferation (Fig.7B). Silencing efficiency of c-Jun is shown in Figure S4. Cumulatively, this data indicate the pro-proliferative effects of HMGB1 are driven by MAPK activation *via* c-Jun transcription factor.

## Discussion

In this study, we investigated the role of HMGB1 and its intracellular signalling pathways in regulating vascular remodelling processes. In IPAH and COPD+PH patients, remodelled pulmonary arteries were associated with HMGB1-positive inflammatory cells. Furthermore, elevated circulating levels of HMGB1 were observed in serum samples from these patients. *In vitro*, we demonstrated a pro-proliferative effect for HMGB1 on human PASMC, an effect that could be blocked using pharmacological or siRNA intervention. Together, our observations indicate a paracrine role for HMGB1 in adversely altering pulmonary homoeostasis *via* activation of c-Jun and consequently vascular remodelling.

Inflammatory cells and mediators have been increasingly implicated in the development and progression of PH 21. HMGB1 has been shown to be elevated in sputum of asthma and COPD patients 7,22 and increased serum levels of HMGB1 were measured in IPAH patients 23. Accordingly, we have shown elevated HMGB1 serum levels in IPAH and COPD+PH patients. HMGB1 can stimulate the production of several pro-inflammatory factors associated with PH pathogenesis, including CCL2, IL-8 and PAI1 24. In human and experimental PH, increased circulatory levels of cytokines such as IL-1β, IL-6 and IL-8 25,26 and chemokines such as CCL2, CCL5 and CXC3CL1 have been observed 27–30. These pro-inflammatory mediators exert direct effects on vascular structural cells, directly altering vessel microenvironment, and circulating inflammatory cells by recruiting them to the vessel wall where they can then release HMGB1. Consistent with this notion, we observed strong immunoreactivity for HMGB1 in inflammatory cells, although other cell types (such as endothelial or smooth muscle cells) could also release HMGB1 and thus contribute to increased circulatory levels in patients with IPAH and COPD+PH. As raised HMGB1 levels were detected in patients with established PH, we cannot say whether HMGB1 is an initiating factor or a consequence of the pro-inflammatory milieu that potentiates the disease phenotype.

Vascular remodelling in IPAH and COPD patients both share some common features such as intimal thickening and medial hypertrophy 9,31, associated with smooth muscle proliferation 32–34. In our study, we demonstrated that HMGB1 increased the proliferation of PAEC as well as migration and proliferation of PASMC *in vitro*. HMGB1 has been shown to have a role in angiogenesis, stimulating the proliferation, chemotaxis and sprouting of murine aortic endothelial cells 35 and also in the proliferation of other cell types, *e.g*. smooth muscle cells 36 and endothelial progenitor cells 37; conversely, a recent publication has indicated no effect of HMGB1 on PAEC proliferation 38. The reason for these discrepancies could be due to the different concentrations of HMGB1 applied (ranging from 1 ng/ml to 1 μg/ml), several publications have described the biphasic proliferation response to HMGB1 37,39. In our experimental set-up, both 1 and 100 ng/ml demonstrated significant pro-proliferative properties. Key pathways that regulate proliferation included MAPK and their downstream signalling molecules. Stimulation of PAEC or PASMC with HMGB1 activated several intracellular signalling pathways (p38, ERK and JNK), although with different kinetics depending on the HMGB1 concentration. Pharmacological inhibition of these signalling kinases revealed a crucial role for JNK and p38 in HMGB1-induced proliferation of human PASMC. This is consistent with a previous report showing that JNK inhibition can attenuate the HMGB1-induced proliferation of hepatic stellate cells 40. In addition, we demonstrated downstream activation of both c-Jun and c-Fos, components of the AP-1 transcription factor complex. The AP-1 complex is an important regulator of both proliferative and inflammatory responses 41. Previously, we have shown that silencing of AP-1 components attenuates PDGF-BB–induced PASMC proliferation 42. Similarly, knockdown of c-Jun prevented the HMGB1-induced proliferation in human PASMC at both 1 and 100 ng/ml concentrations, further supporting the central role of AP-1 complex in vascular remodelling. However, stimulation with high levels of HMBG1 (100 ng/ml) could activate additional processes, as demonstrated by the involvement of ERK, in addition to the p38 and JNK pathways, in regulating PASMC proliferation. This could possibly provide a mechanistic explanation of the biphasic response to HMGB1 proliferative effects using different concentrations. Importantly, the lower HMGB1 concentrations used in our study reflect the circulatory levels observed in IPAH, asthma and COPD patients as measured by us and others 4,5.

Here, we investigated the effects of the pro-inflammatory mediator HMGB1 on vascular cells and its downstream molecular mechanisms that may trigger remodelling in the pulmonary circulation. We have identified that physiological concentrations of HMGB1 are pro-proliferative and act *via* c-Jun activation. HMGB1 represents one common pathway that could contribute to vascular remodelling in different forms of PH. In keeping with this, recent studies have demonstrated that the application of HMGB1-neutralizing antibodies exerted a protective effect in two independent rodent models of PH 23,43, which indicates the potential of HMGB1 inhibition as a novel therapeutic option for PH.

## Supporting Information

**Figure S1** Purity of pulmonary arterial smooth muscle cell isolation as determined by staining against smooth muscle actin (SMA) and smooth muscle myosin heavy chain (SMMHC). Negative staining represents control staining with the omission of primary antibody.

**Figure S2** Expression of extracellular matrix components in (A) human pulmonary arterial smooth muscle cells (PASMC) and (B) human pulmonary arterial endothelial cells (PAEC) as assessed by real-time PCR.

**Figure S3** Densitometric analysis of MAPK and downstream factor activation in (A) human pulmonary arterial smooth muscle cells (PASMC) and (B) human pulmonary arterial endothelial cells (PAEC) following stimulation with 1 or 100 ng/ml HMGB1 for the indicated time-points.

**Figure S4** Real-time PCR analysis of c-Jun expression in PASMC following siRNA treatment, line represents mean, ***P* < 0.01.
