# Supplementary figures and images for: High-mobility group box-1 induces vascular remodelling processes via c-Jun activation
Source: J Cell Mol Med. 2015 Feb 28;19(5):1151–61. doi: 10.1111/jcmm.12519 (PMC4420616; doi:10.1111/jcmm.12519)

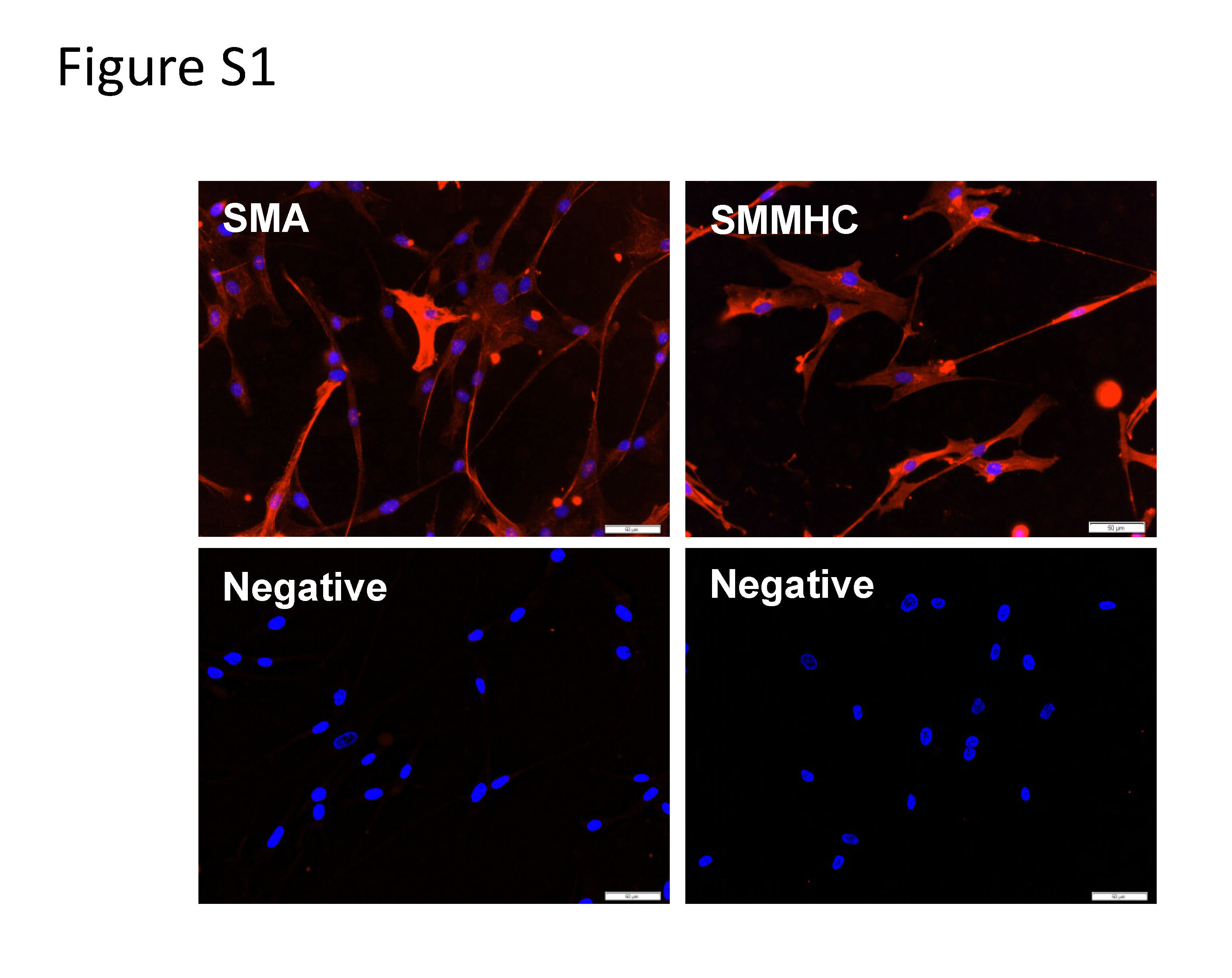

Supplement: Supplementary file 1 [file jcmm0019-1151-sd1.tif]

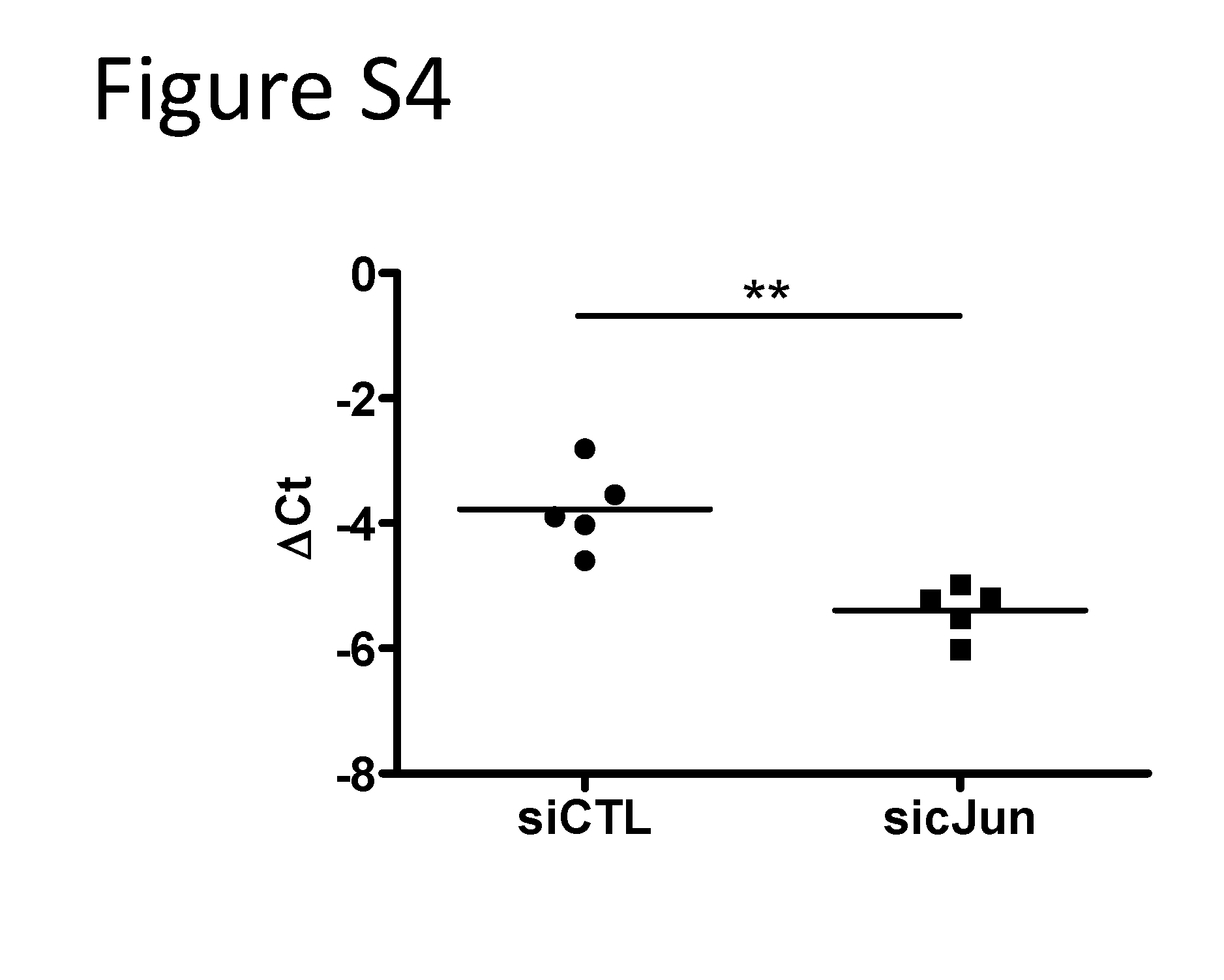

Supplement: Supplementary file 4 [file jcmm0019-1151-sd4.tif]
